# Correlation of Coronary Artery Disease and Left Ventricular Hypertrophy

**DOI:** 10.7759/cureus.17550

**Published:** 2021-08-30

**Authors:** Khizer Khalid, Jaskamal Padda, Dina Ismail, Muhammad Abdullah, Dhriti Gupta, Roshini Pradeep, Warda Hameed, Ayden Charlene Cooper, Gutteridge Jean-Charles

**Affiliations:** 1 Internal Medicine, Jean-Charles (JC) Medical Center, Orlando, USA; 2 Internal Medicine, AdventHealth & Orlando Health Hospital, Orlando, USA

**Keywords:** cad: coronary artery disease, left ventricular hypertrophy (lvh), concentric, eccentric, myocardial infarction, ihd

## Abstract

Ischemic heart disease (IHD) is the leading cause of death worldwide, and it is defined as an imbalance between myocardial oxygen supply and demand. Coronary artery disease (CAD) and left ventricular hypertrophy (LVH) are two common causes of IHD that independently result in myocardial ischemia. CAD decreases myocardial blood and oxygen supply whereas LVH increases myocardial oxygen demand. The coexistence of both CAD and LVH results in a significant increase in oxygen demand while simultaneously lowering oxygen supply.

Since hypertension is a shared predisposing condition for both CAD and LVH, the left ventricular (LV) mass on noninvasive echocardiography can reflect on the severity of coronary artery stenosis. In clinical practice, it can help physicians decide whether to perform invasive cardiac catheterization to visualize the extent of the coronary block. Although, both CAD and LVH are directly proportional to mortality risk, the addition of eccentric LVH can further increase morbidity and mortality due to myocardial infarction. Therefore, the latest management of both the acute and chronic phases of CAD places an increased emphasis on controlling the predisposing factors to prevent or reverse LVH. For example, angiotensin-converting enzyme inhibitors and diuretics reduce LV mass by lowering the cardiac preload and afterload. This article aims to investigate the deleterious effects of the collaboration between CAD and LVH, establish a causal relationship, and explore the new prevention and management strategies.

## Introduction and background

Left ventricular hypertrophy (LVH) is a well-established risk factor for complications like arrhythmias, myocardial infarction, and stroke [1-3]. Coronary artery disease (CAD) is also a broad term that encompasses both the obstructive and non-obstructive parts of the disease and has a lot of risk factors. These risk factors have been divided into modifiable and non-modifiable factors. Non-modifiable factors include gender, age, family history, etc. [4,5] and modifiable factors include hypertension, hyperlipidemia, diabetes mellitus, smoking, obesity, unhealthy diet, and psychosocial conditions [6-8]. One of the major complications attributed to CAD and LVH is ischemic heart disease (IHD). Although LVH has been well studied as a consequence of CAD, there has been a gap in studies regarding LVH as a significant risk factor for CAD. Hence the utility of this article, which combines all the available literature, is to provide a review that gives a much-needed idea about the correlation between CAD and LVH and the incidence of IHD. The further purpose of this review is to comb through the data and show not only an overview of both terms but also to collect evidence to impart how much LVH can impact CAD independent of the other risk factors. The review article starts with the basic definition and perimeters of both the aforementioned terms, followed by epidemiology, the prevalence of CAD and LVH along with the diagnosis and risk factors for both conditions. In the end, the prognosis of both events after viewing them in the same context has been included, and apart from what is already available, possible areas that need further research are discussed.

## Review

CAD

Prevalence

**Figure 1:**
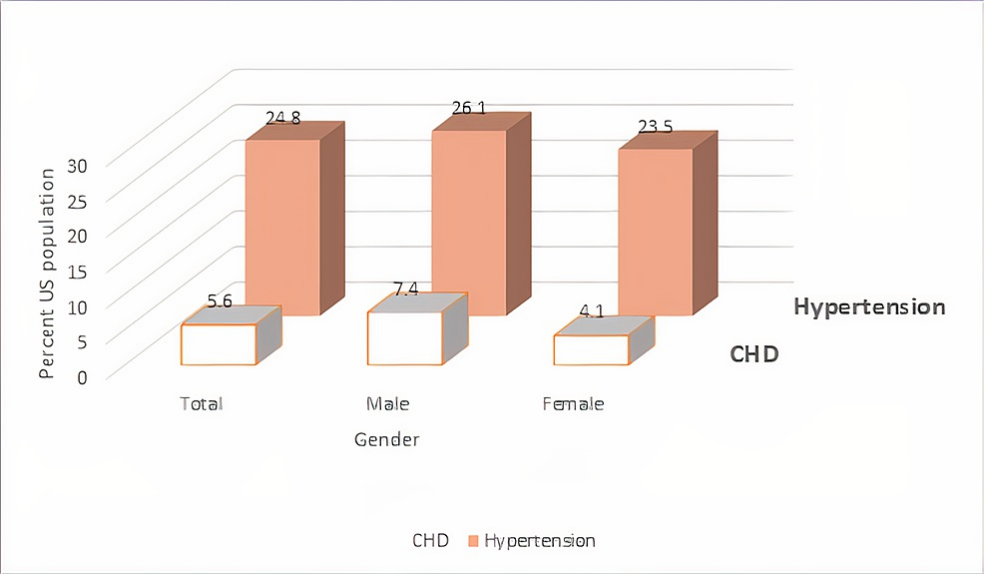
Prevalence of hypertension and coronary heart disease (CHD) by gender. Prevalence of hypertension and CHD in the United States by gender in adults ≥18 years of age, National Health and Nutrition Examination Survey (NHANES), 2015-2018 [10]. The image was created by the author (Dr. Dhriti Gupta, MBBS).

**Figure 2:**
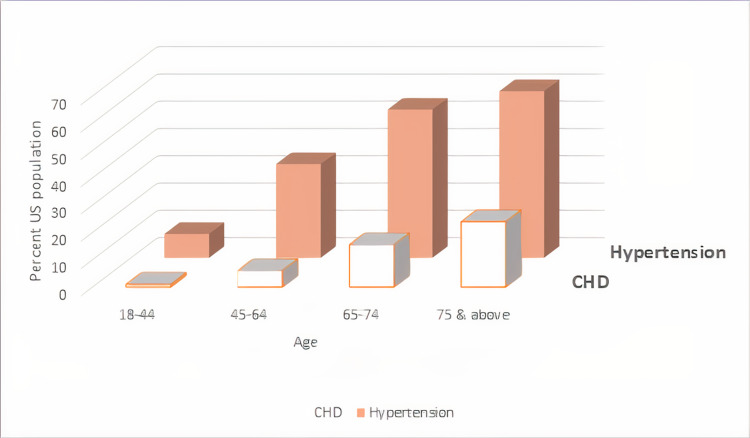
Prevalence of hypertension and coronary heart disease (CHD) by age within the United States, National Health and Nutrition Examination Survey (NHANES), 2015-2018. [10]. The image was created by the author (Dr. Dhriti Gupta, MBBS).

Around 7.2% of American adults ≥20 years of age suffer from CAD. White males have the highest prevalence of CAD (8.6%), followed by Black females (7.2%), Hispanic males (6.8%), and Black males (6.7%). If we consider the acute phase of CAD, an American will experience a myocardial infarction every 40 seconds [9-11]. Prevalence of hypertension and CAD by gender is illustrated in Figure 1, and by age in Figure 2.

Pathophysiology

**Figure 3:**
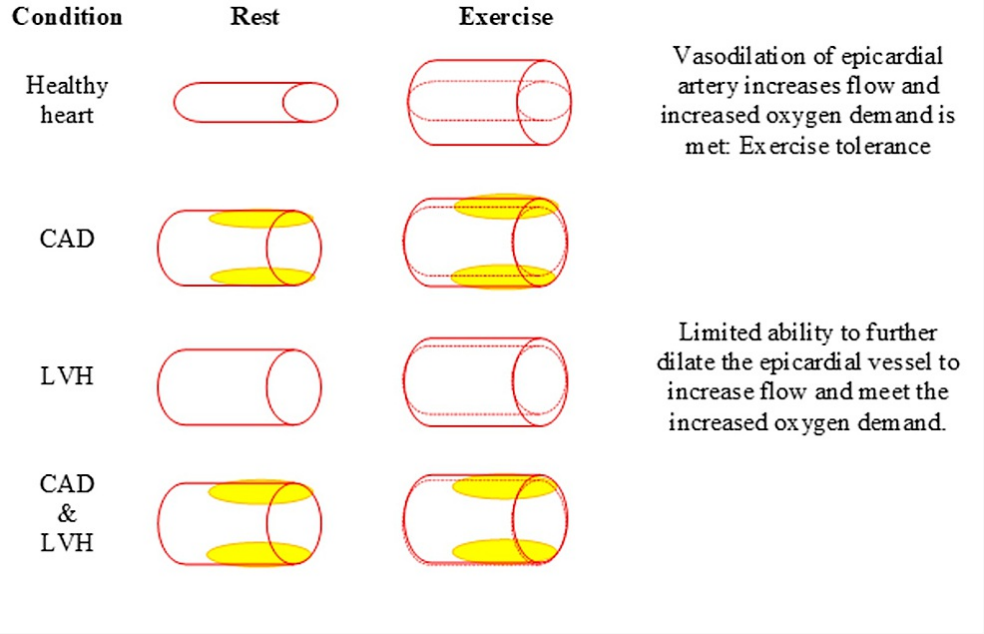
Pathophysiology of exercise intolerance in CAD and LVH. CAD - coronary artery disease, LVH - left ventricular hypertrophy [13]. The image was created by the author (Dr. Dhriti Gupta, MBBS).

CAD is the atherosclerotic narrowing of large epicardial arteries that leads to a decrease in the supply of blood and oxygen to the myocardium. The resulting imbalance between myocardial oxygen demand and supply causes IHD. Predisposing factors include hypertension, dyslipidemia, hyperglycemia, genetic predisposition, etc. which results in atherosclerotic plaque formation within the walls of blood vessels. In obstructive CAD, the atheroma grows inward and causes stenosis of the vascular lumen, decreasing blood flow even at rest [12]. In order to maintain blood flow, these stenosed epicardial arteries remain dilated at rest, therefore limiting their ability to increase the flow to meet increased myocardial oxygen demand during physical exertion. Hypertension, aortic stenosis, and LVH further increases the myocardial oxygen demand at rest leading to severe exercise intolerance. In non-obstructive CAD, on the other hand, the atherosclerotic plaque grows outward but has a very thin fibrous cap making it susceptible to rupture. When the plaque ruptures it leads to thrombus formation and subsequently results in acute obstruction of the lumen causing myocardial infarction (Figure 3) [12,13].

Diagnosis

Although there is an added benefit of immediate revascularization, the invasive conventional coronary angiography is now being bridged by noninvasive functional cardiac testing. Among 898 suspected CAD patients in the CECaT (Cost-Effectiveness of noninvasive Cardiac Testing) study, 20% to 25% avoided invasive angiography using gateway functional tests such as stress echocardiography, single-photon emission computed tomography (SPECT), and magnetic resonance imaging (MRI). SPECT strategy was superior with the highest positive (94%, p = 0.05), least false negatives (31%), and nonsignificant costs. Interestingly, 52% of patients with negative MRI had a subsequent positive angiography and thus showed the largest failure rates [14]. A combination of coronary computed tomographic angiography (CTA) and perfusion (CTP) imaging is more powerful in diagnosing flow-limiting stenosis than CTA or CTP alone. Also, CTP is superior to CTA in females [15].

Prognosis

Physical inactivity, obesity, and smoking are well-established lifestyle-related risk factors of CAD [16]. When patients from OASIS 5 (Fifth Organization to Assess Strategies in Acute Ischemic Syndromes) trial were followed up for six months, those who modified diet, exercised (odds ratio (OR) of 0.57; 95% confidence interval (CI) of 0.36 to 0.89), or stopped smoking (OR: 0.52; 95% CI of, 0.4 to 0.69) had a lowered incidence of repeat myocardial infarction [17]. The RESPONSE (Randomised Evaluation of Secondary Prevention by Outpatient Nurse Specialists) trials demonstrated the added benefit of nurse coordinated counseling on adherence and referral to community-based lifestyle modification programs on improvements of lifestyle-related risk factors [18,19]. The COURAGE (Clinical Outcomes Utilizing Revascularization and Aggressive Drug Evaluation) research group found that adding percutaneous coronary intervention (PCI) to medical therapy had no benefit in stable CAD [20]. In 2013 a meta-analysis of 89,933 patients found that complete revascularization after coronary artery bypass grafting resulted in increased long-term survival when compared to incomplete revascularization (risk ratio: 0.71; p < 0.001). PCI resulted in a majority of the incomplete revascularizations which led to more myocardial infarctions and repeat coronary revascularizations [21].

LVH

According to the Framingham Heart Study, it has been suggested that the presence of features of LVH in electrocardiography is an independent predictor of all-cause mortality [22]. Additionally, 15%-20% of the population was proven to have LVH [23]. LVH is characterized by increased mass in muscle fibers which is secondary to increased stress on the ventricular wall; this compensates for increased wall thickness, leading to improved contractility [24].

Risk Factors

Hypertension and aortic stenosis are the two most common risk factors for LVH. Even without any prevalence of underlying cardiovascular disease, hypertension is an independent risk factor for LVH [23]. The common risk factors for both concentric and eccentric hypertrophy include age, female gender, 24-hour systolic blood pressure greater than 140, body mass index greater than 25. Whereas the risk factors exclusive for eccentric hypertrophy include high-density lipoprotein. Moreover, a drinking history was found as a protective factor for eccentric hypertrophy [25,26]. Studies suggest a 1.5-fold increased risk of LVH in patients with a history of type 2 diabetes mellitus. Interestingly, central obesity had a positive interaction with diabetes mellitus on the development of LVH. In contrast, obesity, in general, does not play a role in the development of diabetes mellitus and LVH [27]. A recent study by Niiranen et al. showed that hypertension age of onset has a minor role compared to the simple presence of hypertension itself in the development of electrocardiographic (ECG) LVH [28]. In this study, hypertension onset at age <40 years association with ECG-LVH was similar to its correlation with hypertension onset at age ≥45 years [28]. This contradicts the previously well-accepted hypothesis of a strong association of hypertension onset age with LVH [28], which was demonstrated by the CARDIA study (Coronary Artery Risk Development in Young Adults), where the onset of hypertension at an age <35 was related to increased LVH (OR of 2.29; 95% CI of 1.36-3.86) compared to hypertension onset at age ≥45 years, which was not related to increased odds of LVH [29].

Diagnosis of LVH

The most widely used tool in the diagnosis of LVH is the ECG due to its feasibility. Also, it can be performed quickly and has high reproducibility. There are 22 conventional criteria for the diagnosis of LVH based on ECG changes. The 12 lead ECG criterion is based on calculating QRS voltage which is widely used in the diagnosis of LVH [30]. Furthermore, a study conducted in the Multi-Ethnic Study of Atherosclerosis (MESA) population suggested that the use of an ECG was similar to an MRI in predicting cardiovascular morbidity and mortality due to LVH [31]. Therefore, the ECG can prove to be beneficial in detecting the etiology of LVH, including valvular disease or cardiomyopathies. ECG uses a linear method of LV mass estimation, using end-diastolic linear measurements of the interventricular septum, LV inferolateral wall thickness, LV internal diameter. These measurements are derived from 2D guided M mode or direct 2D echocardiography. The assumption of the prolate ellipsoid shape of the LV is made, and the calculations are done using Devereux and Reichek "cube" formula [32].

Eccentric and Concentric Hypertrophy

The prevalence of eccentric LVH is high compared to concentric LVH. The presence of eccentric hypertrophy is associated with worse outcomes in patients with acute coronary syndrome. It causes an increase in oxygen demand in the myocardium, making them prone to myocardial infarction [2]. A study conducted by Verma et al. suggests that eccentric hypertrophy causes a decreased ejection fraction whereas the concentric form of LVH does not directly affect ejection fraction but is associated with a higher risk of adverse cardiac events following an ST elevated myocardial infarction (STEMI). Hence, prognosis associated with concentric and eccentric LVH cannot be determined by the infarct size or myocardial salvage but by the underlying mechanism of hypertrophy development [33].

Various studies have suggested that hypertension is the primary etiology in concentric hypertrophy, whereas cardiomyopathy plays a significant role in developing eccentric hypertrophy. Earlier, it had been assumed that heart failure with reduced ejection fraction is characteristic of eccentric hypertrophy, a considerable proportion of individuals with an apparent reduction in LV ejection fraction had concentric remodeling instead. This finding was primarily observed in older hypertensive women. This population in the study did not benefit from beta-blockers, angiotensin-converting enzyme inhibitors, or angiotensin receptor blockers compared to patients with heart failure with reduced ejection fraction due to eccentric hypertrophy who benefited from this management [34].

Morbidity and Mortality

LVH can be a strong predictor of cardiovascular morbidity and mortality in patients without underlying CAD even after adjusting other major cardiovascular risk factors such as age, smoking, obesity, dyslipidemia, blood pressure, and diabetes. A directly proportional relationship is established between LVH and mortality [35]. Moreover, LVH can cause an increase in mortality due to ventricular arrhythmia, and diastolic dysfunction by itself can cause neurohormonal changes predisposing to arrhythmia. Additionally, LVH can be a marker in patients with coronary heart disease who can develop arrhythmia due to atherosclerosis, endothelial dysfunction, or remodeling [1].

Features of eccentric hypertrophy causing increased mortality and recurrent myocardial infarction make patients prone to major adverse cardiac events at higher rates. The calculated hazard ratio of eccentric hypertrophy is 1.08 and 1.56 for all-cause mortality and major adverse cardiac events, respectively [31]. Quinones et al. performed a 2D echocardiogram on patients in the Studies of Left Ventricular Dysfunction (SOLVD). This study was paramount in establishing LV mass estimates associated with determining hospitalization due to cardiovascular causes and all-cause mortality. These results emphasize that pathologic LVH can be the cause of adverse cardiovascular events. Hence, the need arises to focus on drug development targeted to inhibit its growth [36]. 

CAD and LVH

LVH as a Cause of CAD

LVH has been linked to a variety of negative cardiovascular outcomes including death, myocardial infarction, and heart failure, regardless of whether it is detected by an ECG or echocardiography [37,38]. Although there has been much debate about why LVH is such an important risk factor [39,40], the basic mechanisms that predispose patients with LVH to develop atherosclerosis and consequent CAD are not well understood. Abnormalities in the coronary arteries [41], platelets [42], increased blood viscosity [43], and a prothrombotic condition [44] have all been proposed as possible mechanisms. In addition to these variables, which may also lead to a reduction in myocardial oxygen delivery, individuals with LVH have a higher myocardial oxygen demand [40]. Another basic explanation for the link between LVH and CAD is that LVH acts as a noninvasive sensor of the amount of atherosclerosis and CAD since it reflects target organ damage from concomitant risk factors like hypertension. This idea is supported by the fact that LVH is linked to atherosclerosis in other vascular regions, such as the carotid artery [45]. Another developing theory is that LVH on its own acts as a low-level inflammatory condition, as evidenced by recent animal [46] and human research [47,48]. If this is the case, the proinflammatory state linked to LVH could raise the risk of atherosclerosis, CAD, and myocardial infarction.

Diagnosis

The Framingham study has established that echocardiography is significantly more sensitive than ECG in detecting LVH. Unlike ECG LVH, which occurs in just 3.2% of the general population, echographic LVH was a much more common finding in this research population, occurring in 16% to 19% [49]. Ever since, more studies have supported their research evidence with the use of echocardiographic tools, as it provides not only accurate diagnosis of LVH, but more information on the geometry, structural and functional abnormalities, and other hemodynamic variables of the LV [50].

Association of LVH and CAD

The Framingham study, which included 4976 participants, was one of the first to prove the association between LVH and CAD. It has demonstrated that in both sexes, a history of myocardial infarction increases the chance of LVH by more than threefold, whereas angina pectoris without a history of myocardial infarction doubles the risk in males [49].

The LIFE study, which focused on left ventricle structural and functional abnormalities in a large group of hypertensive patients, found that patients with CAD exhibited higher LV cavity dimensions and mass than their control group. Although both groups presented with eccentric LV hypertrophy, patients with CAD had a more significant prevalence of eccentric hypertrophy than concentric hypertrophy or remodeling. Additionally, lower LV systolic function and greater myocardial afterload and peripheral resistance were found in the CAD group. As a result, patients with CAD exhibited reduced cardiac output and index values [50].

Another interesting finding of this study is that patients with symptomatic CAD had about 80% higher LV mass and 20% higher LV wall stress than healthy people. As a result, the estimated myocardial oxygen demand index in patients with CAD was 1.2 times higher than patients without CAD and 2.15 times higher than the average healthy population [50].

A study conducted on participants with stable, treated angina pectoris revealed that LVH diagnosed with echocardiography was highly prevalent in this study population (75%) [51]. Interestingly, even with the presence of a normal office blood pressure or 24-hour ambulatory blood pressure monitoring, LVH was still predominant. What’s more controversial in this study is that in actual practice, the current blood pressure measurement would be a poor indicator of whether a patient has LVH. In fact, the occurrence of “normotensive” LVH patients in CAD is not truly surprising, since other variables like myocardial contractility and hemodynamic volume burden, may play a role in LV mass [51]. This was also supported by Eskerud et al., who found that, independently of hypertension, LVH was associated with the presence of myocardial ischemia [52].

In contrast to the previous study, concentric LVH was the most common LV geometry type (39%) in patients with stable and treated angina [51]. Ghali et al. also reached a similar conclusion with an increase in LV posterior wall and septal thickness and LV mass index [53].

The large Dallas Heart Study established that concentric ventricular hypertrophy (LV wall thickness, concentricity, and indexed LV mass) was independently associated with coronary atherosclerosis measured by coronary artery calcium, which implies the presence of CAD. However, ventricular dilation was not associated with the coronary atherosclerotic burden. These links were especially strong among black patients. Moreover, the study demonstrates a correlation between concentric hypertrophy and higher c-reactive protein levels; however, this link appears to be mediated by LVH risk factors rather than the disease itself. As a result, the synergistic combination of increasing atherosclerotic burden in the presence of concomitant risk factors linked to a proinflammatory environment, patients with concentric LVH may be at greater risk for incident atherothrombotic events [54].

Prognosis of patients with CAD and LVH

After the discussion of the establishment of LVH as a cause of CAD, further consideration is given to the prognosis of LVH and CAD. Data from this well-established study [1] of 1016 patients with stable CAD and LVH was accumulated over a course of 3.5 years of follow-up. After adjustment for confounders like age, sex, diabetes, hypertension, etc, it was found that an increase of 20 units in LV mass increased the adjusted hazard ratio of death by 22% (p=0.001) and the adjusted hazard ratio of sudden or arrhythmic death by 40% (p = 0.004). Total mortality was also higher in patients with LVH (25% vs 11%, p < 0.001), and after adjustment for cardiovascular risk factors, age, sex, and medical therapy, it was found that LVH has a higher ratio of sudden or arrhythmic death (HR 3.1, p = 0.003). Furthermore, LVH in CAD patients had adverse outcomes even after PCI with drug-eluting stents. According to a study [55] of 1704 subjects over a period of eight years, LVH showed a higher incidence of cardiac death as related to controls (4.4% vs 1.2%, log-rank P =0.023, hazard ratio: 3.371, 95% CI: 1.109-10.25; P = 0.032). The result of this study is further backed up by the study by Park et al. [56] which followed outcomes after 30 days of STEMI. In this study [56], after accounting for LVH as an independent factor in STEMI, the all-cause mortality risk was found to be higher (OR, 2.37; 95% CI, 1.096-5.123, p= 0.028). LV mass is independently a powerful indicator of cardiovascular risk [38] even after controlling the risk factor of hypertension and plays a significant role in the development of cardiovascular morbid event risk [57]. The outcome of LVH with CAD can vary from increased risk of myocardial infarction in non-obstructive cardiac disease [52] to sudden cardiac death or arrhythmia [1]. LVH has also been associated with an increased risk of long-term cardiac death [55] and total mortality [1]. However, there is insufficient data on another episode of myocardial infarction in a patient with CAD and a previous myocardial infarction.

Management of LVH in patients with CAD

LVH in patients with CAD should be treated with a combination of lifestyle changes and active management of the comorbidities that cause it. In patients with hypertension, lifestyle measures such as weight loss and sodium restriction have been shown to diminish ECG LVH [58]. Treatment of elevated blood pressure slows the progression of LVH [59]. This can be achieved by using drugs that contain an angiotensin-converting enzyme inhibitor [60]. Angiotensin-converting enzyme inhibitors were the most efficient antihypertensive agents in lowering LV mass, according to a meta-analysis of 109 treatment studies including 2,357 individuals with hypertension [61]. It is well established that angiotensin-converting enzyme inhibitors, beta-blockers, and calcium channel blockers reduced LV mass by reducing wall thickness, while diuretics reduced LV mass by reducing LV volume [61]. However, LV mass was not reduced by alpha-adrenergic blockers [62] or direct-acting vasodilators [63].

In normotensive patients with CAD, very few studies have looked into the management of LVH. It has been suggested that we will need to achieve lower than traditional blood pressure targets (e.g., a systolic blood pressure of 120 mm Hg) or even a personalized blood pressure target level that guarantees full LVH regression in that population [51]. The E4 study examined the possibility of adding an aldosterone blocker (eplerenone, enalapril, and eplerenone/enalapril) [64]. Lastly, trientine, which is a copper chelating agent, has been shown to reduce LVH in diabetic normotensive patients [65].

## Conclusions

As it was thought that LVH would mostly be associated with CAD in hypertensive patients, this review article has demonstrated that this association also exists in normotensive patients. In fact, LVH is an independent factor not only associated with eccentric hypertrophy but also concentric hypertrophy. The intricate pathophysiology of this correlation considered LVH as a pro-inflammatory factor that would promote atherosclerosis in coronary arteries as well as in the carotids. Concerning the management of LVH in CAD patients in the presence of hypertension, the focus is directed on the treatment of elevated blood pressure. However, in normotensive patients, there are no specific recommendations on the management of LVH in this particular population. For this purpose, future studies should be focused on elucidating this aspect of management. Research studies on the pathophysiology responsible for the development of LVH in normotensive patients with established CAD on animal and human models would also be an interesting prospect.
